# A Preliminary Study Comparing Pre-service and In-service School Principals’ Self-Perception of Distributed Leadership Competencies in Relation to Teaching and Managerial Experience

**DOI:** 10.3389/fpsyg.2022.720459

**Published:** 2022-02-17

**Authors:** Gisela Cebrián, Álvaro Moraleda, Diego Galán-Casado, Olvido Andújar-Molina

**Affiliations:** ^1^Department of Pedagogy, Universitat Rovira i Virgili, Tarragona, Spain; ^2^Faculty of Education, Camilo José Cela University, Madrid, Spain; ^3^Facultad de Educación, Universidad Nacional de Educación a Distancia, Madrid, Spain; ^4^Facultad de Educación, Complutense University of Madrid, Madrid, Spain

**Keywords:** leadership, competencies, sustainability, school principals, distributed leadership

## Abstract

So far little are the studies that have focussed on exploring school principals’ self-conception of their distributed leadership competencies in relation to their managerial and teaching experience. To do so, an exploratory research was carried out with a sample of 163 pre-service and in-service school principals studying a Master’s programme in School Management, Innovation and Leadership at a Spanish University. Data were obtained by using an *Ad hoc* questionnaire of 7 units of competence and 5 proficiency levels for each unit, based on an existing rubric to analyse students’ self-conception of their development of leadership competencies. The findings of this preliminary study show statistically significant differences in the self-perception in all dimensions associated to Managerial Experience (ME) and Teaching Experience (TE) in schools. Study participants with ME showed statistically higher levels than those who had non-ME in four of seven dimensions: lead the school organisation, address the needs of the students, manage the organisation of the school organisation, and manage administrative work. Similar results were obtained in relation to TE versus non-TE were statistically significant differences are found in six dimensions: manage pedagogical and didactic resources, attend to the needs of students, manage didactic strategies, manage the organisation of the school organisation, manage the link between the school organisation and the community, and lead the school organisation. This study shows the importance of teaching and professional experience to acquire leadership competencies in education, therefore the school principal should also be a teacher. This preliminary study provides insights into the relevance of providing pre-service or in-service school principals with training and professional development programmes on sustainability distributed leadership that enable them to genuinely engage the school community, develop innovative pedagogies and lead the process of change toward building more sustainable schools.

## Introduction

Educational transformation toward sustainability also requires effective leadership, leaders who are capable of: translating vision into a comprehensive transformative change process; negotiating the change process with the different organisation agents and at the different institutional levels; assisting and including staff and the community; and being decisive and transparent (Fullan, 2003; Scott et al., 2012). Over the last decades, universities and academics have put efforts to develop sustainability competencies’ frameworks and educational interventions, and to embed these mainly in higher education and teacher training (Wiek et al., 2011; Brundiers et al., 2021). However, the existing literature shows that further embedment of sustainability in pre-service teacher education, and specific training and professional development programmes on Education for Sustainable Development (ESD) for teachers and school principals are required to genuinely engage the school community in sustainability and to put in practice the leadership approaches necessary to build sustainable schools (Zachariou et al., 2013; Dyment and Hill, 2015; Ortega-Sánchez et al., 2020).

A wide agreement exists on distributed and transformative leadership as the leadership approaches required to create sustainable schools and to engage teachers and students in sustainability (Algan and Ummanel, 2019; Mogren and Gericke, 2019). Distributed leadership facilitates organisational change as it focuses on school leaders’ democratic and equal participation and collaboration with all school members promoting a sustained organisational development over time (Harris, 2011; Spillane, 2012). Transformational or transformative leadership goes a step further in terms of critical thinking and worldviews’ questioning, as if focuses on reframing existing mental models, attitudes and actions associated with sustainability (Byung-Jik et al., 2018). Despite the acknowledgement of the importance of leadership for achieving the integration of sustainability within schools, no agreed or common ESD leadership framework for schools or educational institutions exists and no research has documented this type of leadership in schools in a systematic way, without transcending good practices or specific case studies (Hallinger and Suriyankietkaew, 2018; Dries Verhelst et al., 2020).

Sustainability leadership research within the education arena is in its infancy (Lambert, 2012). Authors such as Hargreaves and Fink (2006), Davies (2009), and Hargreaves (2009) have developed theoretical frameworks and models to identify the principles and skills required for sustainability leadership. For example, Hargreaves and Fink (2006) built a model with seven key principles of sustainable leadership, namely: *depth* in learning and integrity; *length* referring to endurance and success over time; *breadth* of influence, promoting distribution rather than delegation; addressing *social justice*; developing environmental *diversity* having into account complexity and cohesion; development of human and material *resources* rather than depletion; and *conservation* and activist engagement with the environment through networks and alliances.

The importance of organisational conditions and a leadership approach oriented toward achieving sustainability has been highlighted in the literature (Kadji-Beltran et al., 2013). Therefore, recent literature has also focussed on conducting systematic literature reviews and developing theoretical frameworks on sustainability leadership, acknowledging its importance in creating sustained change and transformation within educational institutions. For example, Hallinger and Suriyankietkaew (2018) used science mapping tools to review 953 Scopus-indexed documents explicitly concerned with sustainable leadership and identified that most of research to date consists of case studies and single company quantitative surveys. Also, Müller et al. (2020) conducted a literature review to develop a conceptual framework, which offers four stages for the integration of sustainability and ESD in a school, identifying practical actions and management strategies. Dries Verhelst et al. (2020) identified the characteristics of the school facilitating ESD effectiveness through a literature review coming up with eight characteristics of an ESD-effective school organisation: sustainable leadership, school resources, pluralistic communication, supportive relations, collective efficacy, adaptability, democratic decision-making and shared vision.

According to Stevenson et al. (2014) sustainable leadership in schools implies: including ESD in the school vision to create holistic change; promoting ESD learning and understanding amongst school staff and teachers in their everyday practices; developing a professional learning community toward ESD; and promoting a whole-institution approach on ESD to facilitate its implementation in the different spheres of action. This implies the promotion of democratic decision-making, empowerment and collaboration amongst the school agents including students, teachers and the local community (Jackson, 2007). Therefore, sustainable leadership includes a leadership approach based on distribution and empowerment and creating whole-school approaches, which can be a challenge in the current education systems, which focus on control and accountability of school principals and teachers (Stevenson, 2007; Kadji-Beltran et al., 2013; Burns et al., 2015).

The existing studies in the education sector exploring school principals’ knowledge, values and skills toward creating sustainable schools have mainly used questionnaires and mixed-method approaches. Algan and Ummanel (2019) used a mixed methods approach to research distributed leadership, organisational happiness and quality of work life in preschools, concluding that school leaders’ behaviours showed the put in practice of distributed leadership. Also, Zachariou et al. (2013) designed a questionnaire to explore principals’ self-reported competence for organising and implementing ESD in schools and their needs in education and training in order to effectively lead sustainable schools. The analysis revealed that school principals in Cyprus are poorly equipped for their new role as leaders of sustainable schools and agents of change, and focussed on exploring suitable forms, content and approaches for their professional development on ESD.

Different tools are being designed and utilised to assess learners’ sustainability competencies. Questionnaires have been commonly used to assess or explore knowledge, attitudes, and behaviours toward sustainability (Kagawa, 2007; Biasutti and Frate, 2017). Qualitative tools such as rubrics, conceptual maps, reflexive diaries and interviews are also being developed as suitable instruments to assess sustainability competencies (García et al., 2017; Sandri et al., 2018). In a recently conducted literature review in this topic (Redman et al., 2021) three types of assessment are identified including self-assessment, observation, and test-based tools, where self-evaluation and assessment tools were underrepresented in relation to others.

While sustainability competencies frameworks have been developed for higher education and educators, no agreed or validated framework exists in relation to sustainability leadership competencies (Cebrián et al., 2020). The operationalisation of sustainability leadership competencies through the establishment of theoretical frameworks and assessment tools remains as a defiance. Therefore, further empirical research is needed through the usage of self-assessment tools to gain evidence on the self-perceived sustainability leadership competencies of school principals and influence of TE and ME that lead to the holistic transformation of educational institutions to embed ESD through distributed leadership and whole-school approaches.

So far little studies have documented in-service and pre-service school principals’ perception in relation the educational leadership qualities and processes to build sustainable schools and how these are developed through TE and ME (Davis et al., 2005; Kadji-Beltran et al., 2013). Whilst they are envisioned as key change agents toward embedding sustainability within schools, for their privileged decision-making position and capacity to influence school organisational conditions (Jackson, 2007; Birney and Reed, 2009).

For this reason, are there statistically significant differences in the school principals’ self-conception of their sustainability leadership competencies in relation to their ME and TE? Our hypothesis is that people with more TE and ME experience will show higher levels of self-perception of sustainable leadership competencies than people without that experience. This preliminary study was conceived as an exploratory research, where 163 pre-service and in-service school principals studying a Master’s programme in School Management, Innovation and Leadership at a Spanish University responded to an *ad hoc* questionnaire.

Thus, the main objective of this research is to prove if there are statistically significant differences in the self-perception of their development of sustainable leadership competencies depending on whether they have TE or not, and in the same way with ME. Along with this, the complementary objective is to see if these differences are maintained or increased depending on the level of TE and ME.

## Materials and Methods

### Sample

The sample population was composed of 163 pre-service and in-service school principals. With a convenience sampling, all participants were recruited as students of the Master’s programme in School Management, Innovation and Leadership at Camilo José Cela University (Madrid, Spain) in the last three academic years. This master’s programme is delivered online, and the students are from different subject areas, and include pre-service and in-service school principals. All the participants have an undergraduate degree related to education. The sample includes master’s students who are professionals with previous experience and already working as school principals (in-service) or with no previous experience as school principals (pre-service). Students were asked whether they would like to complete an on-line questionnaire about their self-conceived leadership competencies.

An intentional non-probability sampling was used, based on voluntary participation, with ages ranging from 22 to 60 years of age [mean age 33.52 years and standard deviation (SD) of 7.4 years], with a sex distribution of 38.7% men and 61.3% women. The sample, see Table 1, was categorised, according to the Teaching Experience (TE) measured in years, differentiating, on the one hand, between the subjects who did have experience in and those who did not have it, and, on the other hand, between four levels of experience: none (0 years), low (1–4 years), medium (5–9 years) and wide (>9 years). Similarly, in the same table, it was done with the classification on Managerial Experience (ME) (previous professional experience as school principals) measured in years.

**TABLE 1:** Distribution of participants by levels of teaching and managerial experience.

		Level of teaching experience						
		Total	No	Yes	None	Low	Medium	Wide
Level of managerial experience	Total	163	31	132	31	40	41	51
	No	100	31	69	31	33	24	12
	Yes	63	0	63	0	7	17	39
	None	69	31	69	31	33	24	12
	Low	37	0	37	0	7	14	16
	Medium	14	0	14	0	0	2	12
	Wide	12	0	12	0	0	1	11

### Instruments

An *Ad hoc* self-reporting questionnaire formed by 35 items (grouped in 7 dimensions) was designed to analyse pre-service and in-service school principals’ perceptions –self-conception of the development of sustainability leadership competencies. The psychometric properties of the test had satisfactory values for the total score of the scale: internal consistency reliability, Cronbach’s α value of 0.66. The instrument, a scale like Likert 1-5, has evaluated 7 of the central elements in terms of leadership and management of education centres such as: management of pedagogical and didactic resources, attention to the needs of students, management of didactic strategies, managing the organisation of the school organisation, managing the administrative work, managing the connection of the school organisation with the community, and leadership of the school organisation.

**FIGURE 1:**
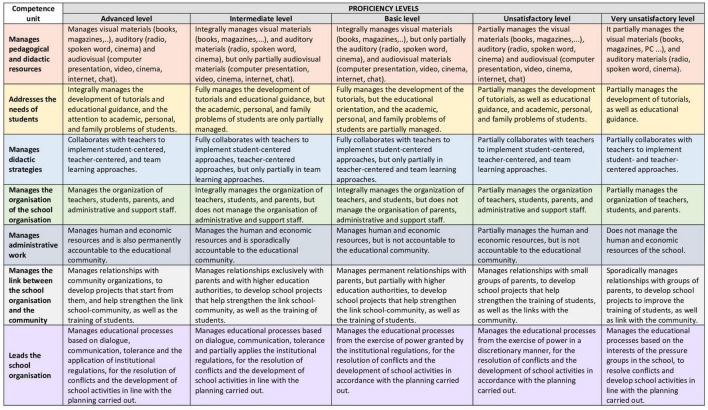
Competence units and proficiency levels of distributed leadership competencies’ framework. Adapted from Mejía Capaza (2015).

As our interest was to explore pre-service and in-service school principals’ self-conception of their sustainability leadership competencies, we adapted an existing self-assessment tool on leadership competencies focussed on distributed leadership developed by Mejía Capaza (2015), which includes 7 units of competence and 5 levels of acquisition/proficiency levels for each unit: advanced, intermediate, basic, unsatisfactory and very unsatisfactory (Figure 1). Study participants had to self-rate their perceived level of proficiency for each unit of competence.

### Design

An *ex post* facto research design has been developed in this preliminary study, the type of research that is applied when looking for the causes and awareness of a phenomenon that cannot occur because it has already happened (Campbell and Stanley, 1963; Fox, 1981; Kerlinger, 1987; Mateo, 1997).

### Procedure

The main analysis focussed on assessing whether more experienced teachers and managers have a perception that their management and leadership intervention in schools increases over the years. The participants completed the questionnaire that included their informed consent in order to be involved in the study. They received no compensation for their participation. Their decision to participate was voluntary and anonymity and confidentiality were guaranteed with regard to data collection and processing. The study was carried out in accordance with the Declaration of Helsinki Ethics.

### Data Analysis

With an anonymous and confidential data collection and treatment, the data analysis was performed using version 26.0 of the SPSS software. Prior to data exploration, the assumption of normality was verified by Kolmogorov-Smirnov (K-S) and the Shapiro-Wilk test. According to the results, non-parametric statistics were used for data analysis, specifically the comparison of Mann-Whitney *U* test and the Kruskal-Wallis *H* test, both with the rank-biserial correlation (*r*_bis_) used as measured effect size estimator.

## Results

The descriptive statistics (mean and SD) of the dimensions, differentiating no/yes and none/low/medium/wide, are shown in Table 2 for TE, and in Table 3 for ME.

**TABLE 2:** Descriptive statistics (Mean and SD) by dimensions based on teaching experience.

	No		Yes		None		Low		Medium		Wide	
	Mean	SD	Mean	SD	Mean	SD	Mean	SD	Mean	SD	Mean	SD
Manages pedagogical and didactic resources	3.55	1.12	4.14	0.98	3.55	1.12	3.90	1.13	4.15	1.04	4.31	0.76
Addresses the needs of students	3.58	1.18	4.25	0.94	3.58	1.18	3.98	1.05	4.24	1.07	4.47	0.67
Manages didactic strategies	3.29	1.16	4.01	0.93	3.29	1.16	3.85	1.00	4.02	1.01	4.12	0.79
Manages the organisation of the school organisation	2.97	1.20	3.33	1.37	2.97	1.20	2.75	1.37	3.34	1.41	3.76	1.19
Manages administrative work	2.61	1.23	2.99	1.50	2.61	1.23	2.68	1.42	2.98	1.62	3.25	1.43
Manages the link between the school organisation and the community	2.87	1.23	3.50	1.24	2.87	1.23	3.15	1.29	3.49	1.34	3.78	1.06
Leads the school organisation	2.97	1.02	3.95	1.13	2.97	1.02	3.65	1.27	3.71	1.19	4.39	0.80

**TABLE 3:** Descriptive statistics (Mean and SD) by dimensions based on managerial experience.

	No		Yes		None		Low		Medium		Wide	
	Mean	SD	Mean	SD	Mean	SD	Mean	SD	Mean	SD	Mean	SD
Manages pedagogical and didactic resources	3.97	1.07	4.11	0.97	3.97	1.07	3.97	1.07	4.21	0.70	4.42	0.90
Addresses the needs of students	3.99	1.09	4.33	0.88	3.99	1.09	4.22	1.00	4.50	0.65	4.50	0.67
Manages didactic strategies	3.80	1.05	3.98	0.94	3.80	1.05	4.00	0.97	4.07	0.62	3.83	1.19
Manages the organisation of the school organisation	2.90	1.34	3.83	1.14	2.90	1.34	3.59	1.19	3.86	1.10	4.50	0.80
Manages administrative work	2.64	1.33	3.37	1.54	2.64	1.33	3.24	1.67	3.43	1.45	3.67	1.23
Manages the link between the school organisation and the community	3.24	1.30	3.60	1.17	3.24	1.30	3.51	1.30	3.64	0.74	3.83	1.19
Leads the school organisation	3.44	1.20	4.29	0.92	3.44	1.20	4.19	1.08	4.21	0.70	4.67	0.49

As the descriptive statistics show, in all dimensions, whether on TE or on ME, the average values are higher if they have experience, which also always increases over the years. In relation to the aim of exploring if experienced teachers and school principals have a perception that their management and leadership intervention in schools increases over time, a non-parametric comparison was made using the Mann-Whitney *U* test.

Regarding the TE, we found statistically significant differences in 5 of the 7 dimensions, with moderate effect size (*r*_bis_ = 0.300), always with better mean rank in the people with such experience. In particular, the differences are significant in the following dimensions: manages pedagogical and didactic resources (*p* = 0.005, *r*_bis_ = 0.270), addresses the needs of students (*p* = 0.002, *r*_bis_ = 0.300), manages didactic strategies (*p* = 0.001, *r*_bis_ = 0.324), manages the link between the school organisation and the community (*p* = 0.013, *r*_bis_ = 0.247), and leads the school organisation (*p* = 0.000, *r*_bis_ = 0.414). The results are provided in Table 4 for TE.

**TABLE 4:** Results of Mann-Whitney *U* test teaching experience vs. no teaching experience in dimensions.

	Teaching experience	*N*	Mean rank	Sum of ranks	*U*	*P*	*r*_bis_
Manages pedagogical and didactic resources	No	31	61.95	1920.50	1424.50	0.005*	0.270
	Yes	132	86.71	11445.50			
Addresses the needs of students	No	31	60.26	1868.00	1372.00	0.002*	0.300
	Yes	132	87.11	11498.00			
Manages didactic strategies	No	31	58.81	1823.00	1327.00	0.001*	0.324
	Yes	132	87.45	11543.00			
Manages the organisation of the school organisation	No	31	70.60	2188.50	1692.50	0.126	0.139
	Yes	132	84.68	11177.50			
Manages administrative work	No	31	72.06	2234.00	1738.00	0.182	0.137
	Yes	132	84.33	11132.00			
Manages the link between the school organisation and the community	No	31	63.48	1968.00	1472.00	0.013*	0.247
	Yes	132	86.35	11398.00			
Leads the school organisation	No	31	48.56	1505.50	1009.50	0.000*	0.414
	Yes	132	89.85	11860.50			

*Significations p < 0.05.

In the same way, regarding the management experience, statistically significant differences were found, with low effect size (*r*_bis_ = 0.100), always with better scores in the experienced subjects, in 4 of the 7 dimensions: addresses the needs of students (*p* = 0.048, *r*_bis_ = 0.169), manages the organisation of the school organisation (*p* = 0.000, *r*_bis_ = 0.350), manages administrative work (*p* = 0.002, *r*_bis_ = 0.246), and leads the school organisation (*p* = 0.000, *r*_bis_ = 0.369). The results are provided in Table 5 for managerial experience.

**TABLE 5:** Results of Mann-Whitney *U* test managerial experience vs. no managerial experience in dimensions.

	Managerial experience	*N*	Mean rank	Sum of ranks	U	*P*	*r*_bis_
Manages pedagogical and didactic resources	No	100	79.99	7998.50	2948.50	0.467	0.068
	Yes	63	85.20	5367.50			
Addresses the needs of students	No	100	76.61	7660.50	2610.50	0.048*	0.169
	Yes	63	90.56	5705.50			
Manages didactic strategies	No	100	79.23	7922.50	2872.50	0.321	0.090
	Yes	63	86.40	5443.50			
Manages the organisation of the school organisation	No	100	69.68	6967.50	1917.50	0.000*	0.350
	Yes	63	101.56	6398.50			
Manages administrative work	No	100	72.94	7294.00	2244.00	0.002*	0.246
	Yes	63	96.38	6072.00			
Manages the link between the school organisation and the community	No	100	76.94	7694.00	2644.00	0.076	0.144
	Yes	63	90.03	5672.00			
Leads the school organisation	No	100	68.67	6866.50	1816.50	0.000*	0.369
	Yes	63	103.17	6499.50			

*Significations p < 0.05.

To complement these results, the hypothesis contrast was supplemented by categorising into four levels (None/Low/Medium/Wide) of TE and ME, using the non-parametric comparison of the Kruskal-Wallis *H* test, which confirmed the vast majority of results.

In the case of TE, see Table 6, it increases the statistically significant differences to 6 of 7 dimensions. Only in dimension manages administrative work, no significant differences are found, in the rest there were with low effect size (*r*_bis_ = 0.100): manages pedagogical and didactic resources (*p* = 0.016, *r*_bis_ = 0.215), addresses the needs of students (*p* = 0.002, *r*_bis_ = 0.267), manages didactic strategies (*p* = 0.009, *r*_bis_ = 0.232), manages the organisation of the school organisation (*p* = 0.002, *r*_bis_ = 0.274), manages the link between the school organisation and the community (*p* = 0.008, *r*_bis_ = 0.235), and leads the school organisation (*p* = 0.000, *r*_bis_ = 0.436). As already mentioned, and can be seen in the mean ranks, in all dimensions the trend over the years is upward, that is, a greater self-perception.

**TABLE 6:** Results of Kruskal-Wallis *H* test level of teaching experience in dimensions.

	Teaching experience	*N*	Mean rank	*H*	df	*P*	*r*_bis_
Manages pedagogical and didactic resources	None	31	61.95	10.342	3	0.016*	0.215
	Low	40	77.65				
	Medium	41	88.11				
	Wide	51	92.69				
Addresses the needs of students	None	31	60.26	14.432	3	0.002*	0.267
	Low	40	74.48				
	Medium	41	89.62				
	Wide	51	94.99				
Manages didactic strategies	None	31	58.81	11.594	3	0.009*	0.232
	Low	40	80.50				
	Medium	41	89.22				
	Wide	51	91.47				
Manages the organisation of the school organisation	None	31	70.60	15.029	3	0.002*	0.274
	Low	40	64.99				
	Medium	41	85.27				
	Wide	51	99.65				
Manages administrative work	None	31	72.06	5.414	3	0.144	0.123
	Low	40	74.01				
	Medium	41	84.16				
	Wide	51	92.57				
Manages the link between the school organisation and the community	None	31	63.48	11.732	3	0.008*	0.235
	Low	40	73.74				
	Medium	41	86.04				
	Wide	51	96.49				
Leads the school organisation	None	31	48.56	33.366	3	0.000*	0.436
	Low	40	78.31				
	Medium	41	79.41				
	Wide	51	107.29				

*Significations p < 0.05.

In turn, regarding the ME, differentiated by the four levels, see Table 7, statistically significant differences are maintained in 3 of the 7 dimensions with low effect size (*r*_bis_ = 0.100), always with higher self-perceptions when there is more experience. The dimensions are manages the organisation of the school organisation (*p* = 0.000, *r*_bis_ = 0.355), manages administrative work (*p* = 0.013, *r*_bis_ = 0.221), and leads the school organisation (*p* = 0.000, *r*_bis_ = 0.367).

**TABLE 7:** Results of Kruskal-Wallis *H* test level of managerial experience in dimensions.

	Managerial experience	*N*	Mean Rank	*H*	df	*P*	*r*_bis_
Manages pedagogical and didactic resources	None	100	79.99	2.333	3	0.506	0.063
	Low	37	80.00				
	Medium	14	86.39				
	Wide	12	99.83				
Addresses the needs of students	None	100	76.61	4.635	3	0.201	0.100
	Low	37	86.57				
	Medium	14	96.11				
	Wide	12	96.42				
Manages didactic strategies	None	100	79.23	1.152	3	0.764	0.111
	Low	37	87.41				
	Medium	14	87.86				
	Wide	12	81.63				
Manages the organisation of the school organisation	None	100	69.68	23.095	3	0.000*	0.355
	Low	37	93.22				
	Medium	14	102.68				
	Wide	12	126.00				
Manages administrative work	None	100	72.94	10.794	3	0.013*	0.221
	Low	37	92.53				
	Medium	14	98.43				
	Wide	12	105.88				
Manages the link between the school organisation and the community	None	100	76.94	3.770	3	0.287	0.071
	Low	37	87.28				
	Medium	14	89.32				
	Wide	12	99.33				
Leads the school organisation	None	100	68.67	24.591	3	0.000*	0.367
	Low	37	100.34				
	Medium	14	96.50				
	Wide	12	119.67				

*Significations p < 0.05.

From an overview, putting together the four analyses carried out previously, the general results of this research reflect statistically significant differences in the self-perception of participation in all dimensions, having higher values in the participants with more TE and ME.

## Discussion

The results of this preliminary study are in line with previous studies such as the one carried out by Coggins and McGovern (2014), where it was concluded that educational leadership significantly contributes to the improvement of the outcomes of teaching and learning processes, also empowering teachers to formulate improvements to increase the effectiveness of the learning environment and student tracking. Another study conducted by Ross and Cozzens (2016) reinforces this premise, highlighting the importance of leadership support when assessing the diversity of teachers’ ideas and opinions. In turn, the most effective school principals from a leadership-centred approach influence the school climate and culture through teacher collaboration and professional development (Louis et al., 2010). Along the same lines, previous studies have shown that educational leaders must recognise and assume a shared responsibility that goes beyond the intellectual and educational development of students, putting in place a real commitment to favour and focus on their personal, social physical and emotional development (Arhipova et al., 2018; Ghirmai Jambo and Hongde, 2020). Likewise, leadership must be understood as a compendium between the characteristics or personality traits and the specific skills for its execution and development (Amanchukwu et al., 2015). This implies not only a specific training and professional development programmes, but also the mobilisation of several intra- and inter-personal competencies to understand and act on the educational needs present in the school.

Although there are studies such as the one developed by Pont et al. (2008), where it is stated that the practice of school leadership requires specific skills and competencies that may not have been developed exclusively with TE, the results of this study and the current conditions of systemic change within educational environments require school principals’ holistic understanding and specific competencies, that are gained through TE and ME. This is in line also with the study conducted by Ortega-Sánchez et al. (2020) that stressed the importance of developing school policies and teaching practice toward the inclusion of contemporary social problems in the curriculum at all education levels holistically.

Leadership competencies include the management of different processes such as educational, strategic, operational, interpersonal and intrapersonal together with reflective practice and continuous learning (Trakšelys et al., 2016). Precisely this continuous learning is a factor that is closely related to professional experience, which allows school principals to have the ability not only to manage external pedagogical axes for the benefit of student learning, but also the development of skills that involve listening and intervening in specific social circumstances, understanding individual subjectivities, and being resilient showing the ability to respond efficiently to unpredictable events (Leithwood et al., 2008; Male and Palaiologou, 2015). These are achieved through the professional experience and knowing the functioning, characteristics and needs of the recipients of the educational processes.

The findings of this study are in line with previous authors such as Escamilla (2006), who affirms that school principals are trained and developed through specific leadership training courses, and also nurtured from their role as teachers and with the daily practice and the experience gained in the school management over the years. These ideas are complemented by Boliìvar (2011), who highlights that the managerial experience and real practice have a critical role play in helping school leaders develop leadership competencies. Davis et al. (2005) also emphasise the importance of applying skills, knowledge and problem solving in authentic environments to achieve an effective managerial and leadership role has also been highlighted. Therefore, as the results from this study show, having previous teaching experience and learning-by-doing through management experience is critical to the development of distributed leadership competencies amongst school principals that can in turn contribute to create more sustainable organisations.

## Conclusion

The existing literature in the area of sustainability education clearly highlights the key role that school leaders play in creating sustainable schools and empowering individuals through education to promote active change agents for the social transformation of societies toward more sustainable, equitable and socially just patterns. This requires effective leaders that are capable of fostering distributed leadership within their organisations where equal participation and collaboration between all school members and stakeholder groups is required.

The aims of this research were to explore the school principals’ self-perception of their development of distributed leadership competencies in relation to previous ME and TE that can lead to creating sustainable schools and embedding ESD holistically within organisations. The findings of this preliminary study clearly show statistically significant differences in relation to the self-perceived development of distributed leadership competencies and previous TE and ME. Therefore, this study shows the importance of professional experience to acquire distributed leadership competencies, therefore the school principal should also be a teacher.

This study provides insights into the relevance of providing pre-service and in-service school principals with training and professional development programmes on sustainability and distributed leadership that enable them to genuinely engage the school community in ESD, develop innovative pedagogies and lead the process of change toward building more sustainable schools.

However, this preliminary study has several limitations, such as the low knowledge of sociodemographic, and professional (ME and TE) variables of the sample. Also, the lack of knowledge of the socio anagraphical variables of the subjects involved, that could have influenced the results obtained. Therefore, the relationship between both should be explored in further empirical research. It should be taken into consideration that the findings of this research explain a specific reality and context in a determined moment, therefore, the influence of contextual, political, educational and socio-cultural factors need to be further explored through cross-sectional studies involving different regions and countries.

Also, further empirical research is needed, beyond the *ex post* facto design with a Likert self-reporting scale in a non-randomised sample, that allows conducting experimental and longitudinal studies with a validated instrument, in terms of reliability and validity (the questionnaire has not been validated through an expert judgement), and a higher sample and number of school principals involved to determine how these leadership competencies are acquired over time and the organisational conditions and factors influencing their development.

Based on the research conducted, the authors suggest 3 pathways for further research and practice that will enhance the development of distributed leadership competencies toward embedding sustainability within educational institutions:

• Conduct longitudinal and cross-sectional studies using summative, formative and self-assessment tools that provide evidence of the development of sustainability distributed leadership competencies as they develop over time and the influence that contextual and organisational factors have.

• Operationalise sustainability distributed leadership competencies as constructs in the design and development of statistically validated assessment tools that guarantee the reliability and quality of the results.
• Develop clear learning and professional development pathways for school principals and leaders through in-service and pre-service leadership professional development programmes oriented to the development of distributed leadership in schools toward sustainability.

## Data Availability Statement

The original contributions presented in the study are included in the article/supplementary material, further inquiries can be directed to the corresponding author.

## Ethics Statement

The studies involving human participants were reviewed and approved by Universidad Camilo José Cela. The participants provided their written informed consent to participate in this study.

## Author Contributions

GC, ÁM, and DG-C contributed to conception and designed of the study. GC organised the database and wrote the first draft of the manuscript. ÁM performed the statistical analysis. GC, ÁM, DG-C, and OA-M wrote sections of the manuscript. All authors contributed to manuscript revision, read, and approved the submitted version.

## Conflict of Interest

The authors declare that the research was conducted in the absence of any commercial or financial relationships that could be construed as a potential conflict of interest.

## Publisher’s Note

All claims expressed in this article are solely those of the authors and do not necessarily represent those of their affiliated organizations, or those of the publisher, the editors and the reviewers. Any product that may be evaluated in this article, or claim that may be made by its manufacturer, is not guaranteed or endorsed by the publisher.
